# Supplementary material for: Nuclear to cytoplasmic transport is a druggable dependency in MYC-driven hepatocellular carcinoma
Source: Nat Commun. 2024 Feb 1;15:963. doi: 10.1038/s41467-024-45128-y (PMC10834515; doi:10.1038/s41467-024-45128-y)
Supplement: Supplementary file 1 — Supplementary Information [file 41467_2024_45128_MOESM1_ESM.pdf]

Supplementary Figure 1

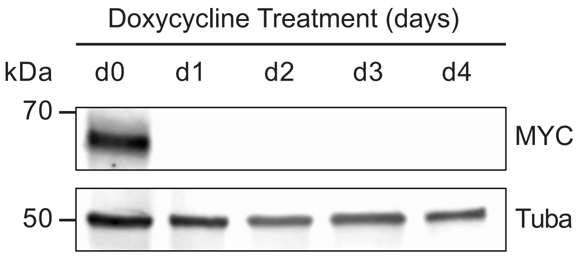

Supplementary Fig 1. Regulation of MYC expression by the Tet system in EC4 cell line.
EC4 cells were treated with doxycycline (100 ng/ml) in two independent experiments for four days and examined for MYC expression by Western blot analysis (representative Western blot is shown). alpha-Tubulin (Tuba) was used as a loading control. Source data are provided as a Source Data file.

Supplementary Figure 2

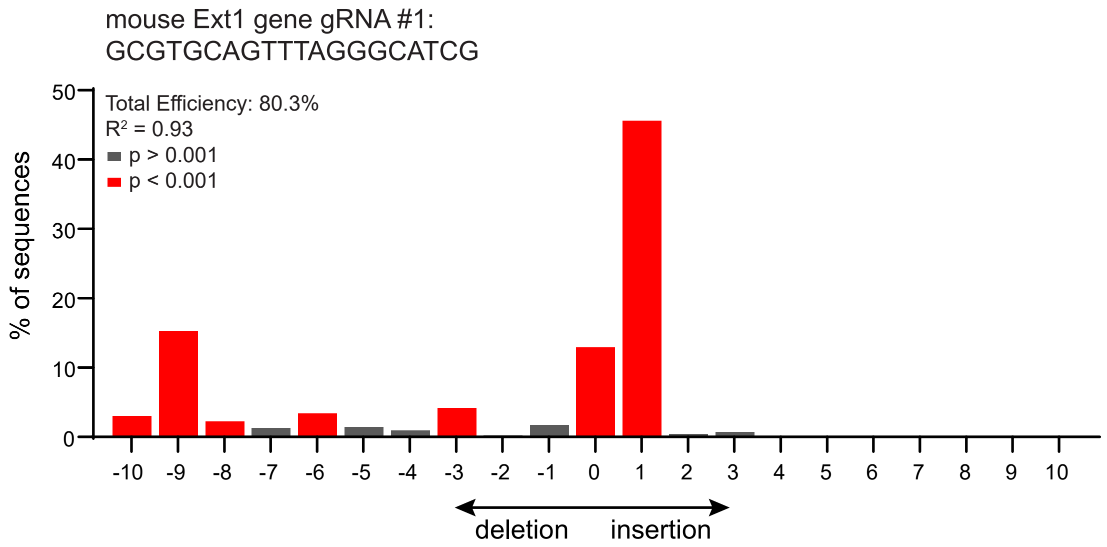

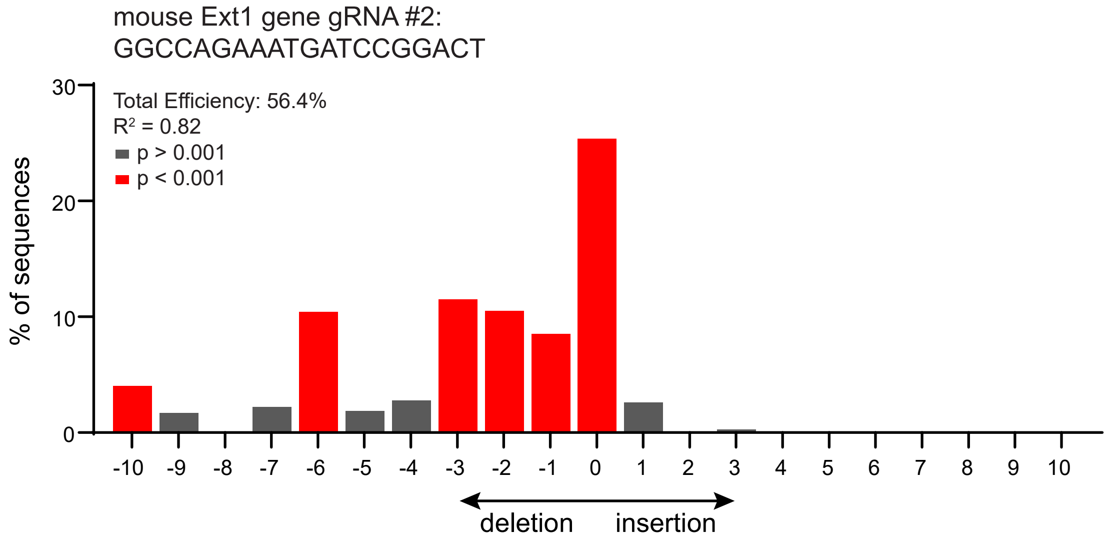

Supplementary Fig 2. Mutation efficiency of mouse Ext1 gene in EC4 cells by two gRNAs. Two gRNAs targeting the mouse Ext1 gene were delivered to EC4 cells with stable Cas9 nuclease expression via lentiviral infection. Cells were examined for Ext1 mutations via PCR two weeks after infection. The predominant types of insertions and deletions in the Ext1 genomic DNA were deconvoluted using the Tracking of Indels by Decomposition (TIDE; https://tide.nki.nl/) algorithm. The two gRNAs induced 80.3% and 56.4% Ext1 mutations, respectively. Exact p values are provided in the source data file. TIDE and statistical methods used were previously reported by Brinkman et al, Nucl. Acids Res. 2014. Source data are provided as a Source Data file.

Supplementary Figure 3

a

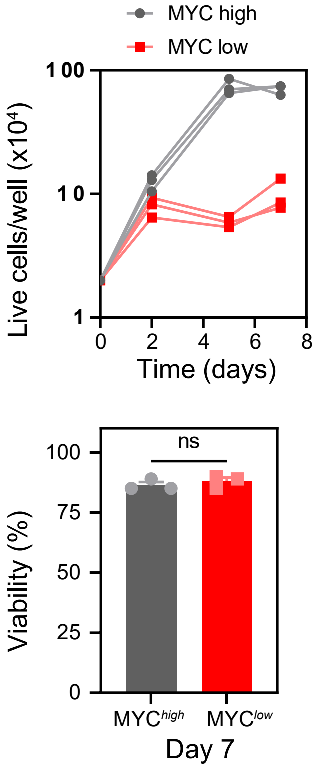

b

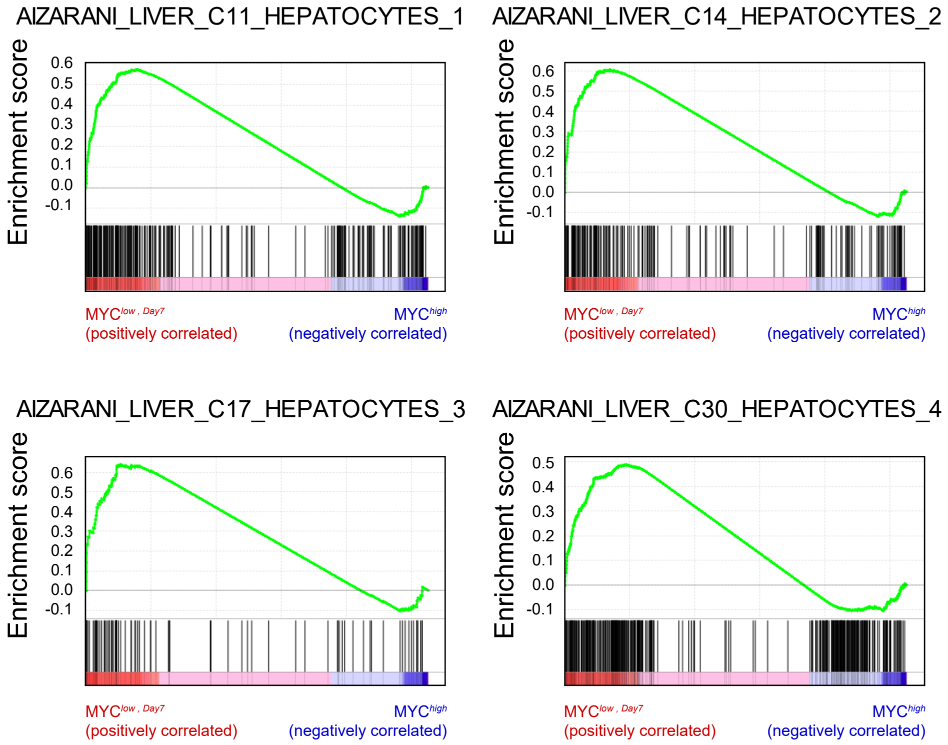

c

Gene set	NES	p-value	FDR q-value
AIZARANI_LIVER_C11_HEPATOCYTES_1	1.42	0.002	0.004
AIZARANI_LIVER_C14_HEPATOCYTES_2	1.48	0.000	0.004
AIZARANI_LIVER_C17_HEPATOCYTES_3	1.43	0.013	0.007
AIZARANI_LIVER_C30_HEPATOCYTES_4	1.24	0.019	0.050

d

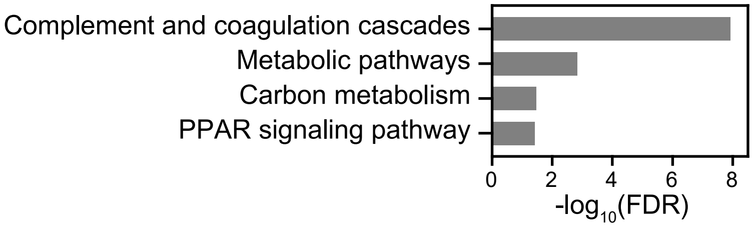

e

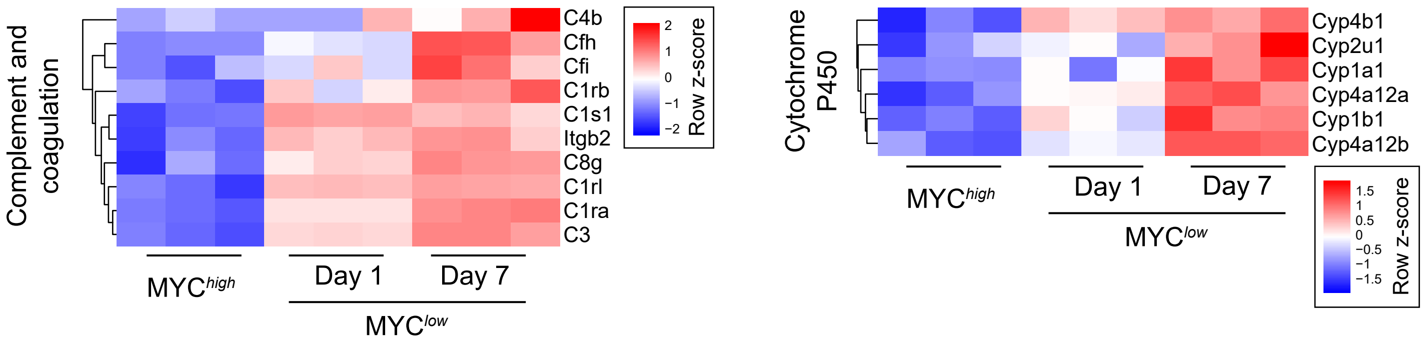

Supplementary Fig 3. Differentiation phenotype of EC4 cell line upon downregulation of transgenic MYC expression. a, Proliferative capacity and viability upon MYC downregulation (n = 3 biologically independent replicates/wells in one experiment). Bar chart shows means, error bars are SEM. **b,c,** Gene set enrichment analysis of orthologs of human hepatocyte genes (Azarani et al., 2019) in mRNA expression data sets of EC4 MYC^{low} (Day7) compared to MYC^{high} cells; FDR is adjusted for multiple hypothesis testing. **d,** KEGG pathway analysis of hepatocyte genes upregulated in MYC^{low} (Day7) EC4 cells. **e,** Heat maps of representative hepatocyte genes upregulated in MYC^{low} (Day1 and Day7) EC4 cells involved in complement and coagulation or cytochrome p450-mediated metabolism. For this figure, source data are provided as a Source Data file.

Supplementary Figure 4

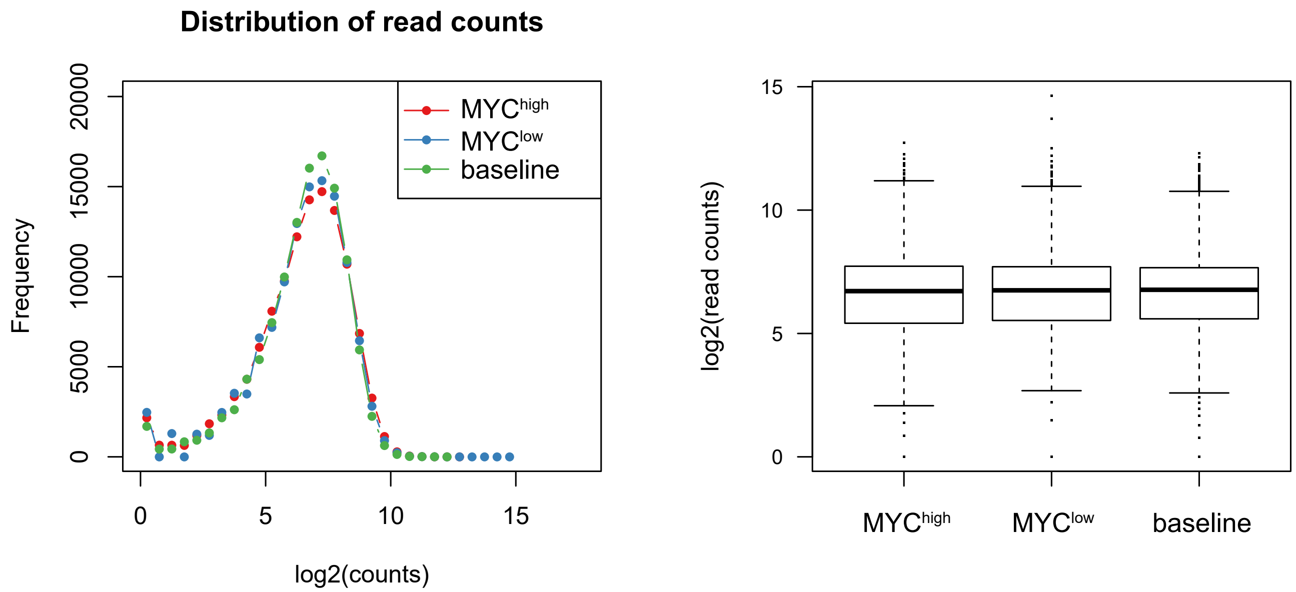

Supplementary Fig 4. Frequency distribution of gRNA read counts.
Distribution of read counts was assessed as a quality control metric to confirm consistent gRNA abundance distribution between samples. The frequencies of all gRNA read counts were determined and visualized by MAGeCK-VISPR in three conditions (MYC^{high}, MYC^{low}, and baseline; one sample per condition). Similar distributions across samples allowed for sample comparison. Right panel: Box center is median, box is 25-75 percentile, whiskers represent data points within 1.5-times interquartile range.

Supplementary Figure 5

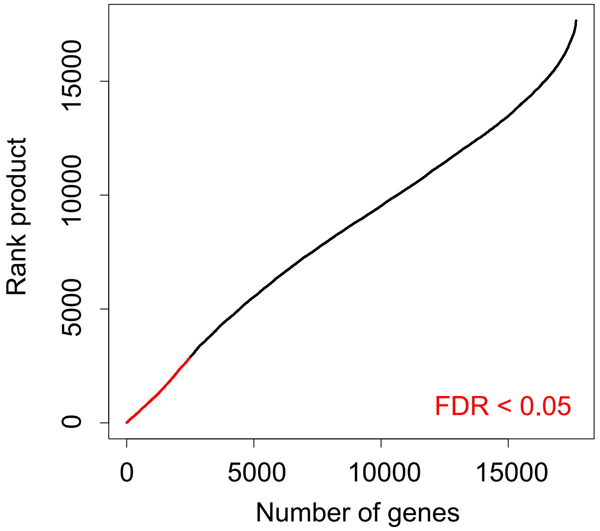

Supplementary Fig 5. Rank product analysis of gene essentiality in human liver cancer cell lines.

Essentiality scores from genome-wide CRISPR essentiality screen in the cancer cell line encyclopedia (CCLE) of 13 human liver cancer cell lines (see Supplementary Table 3) were ranked by rank product analysis using the R package Rankprod (Carratore et al., 2017; Hong et al., 2006). Genes that showed significant (FDR < 0.05) negative scores across the 13 cell lines were defined as essential genes in human HCC (FDR from one-sided Permutation test).

Supplementary Figure 6

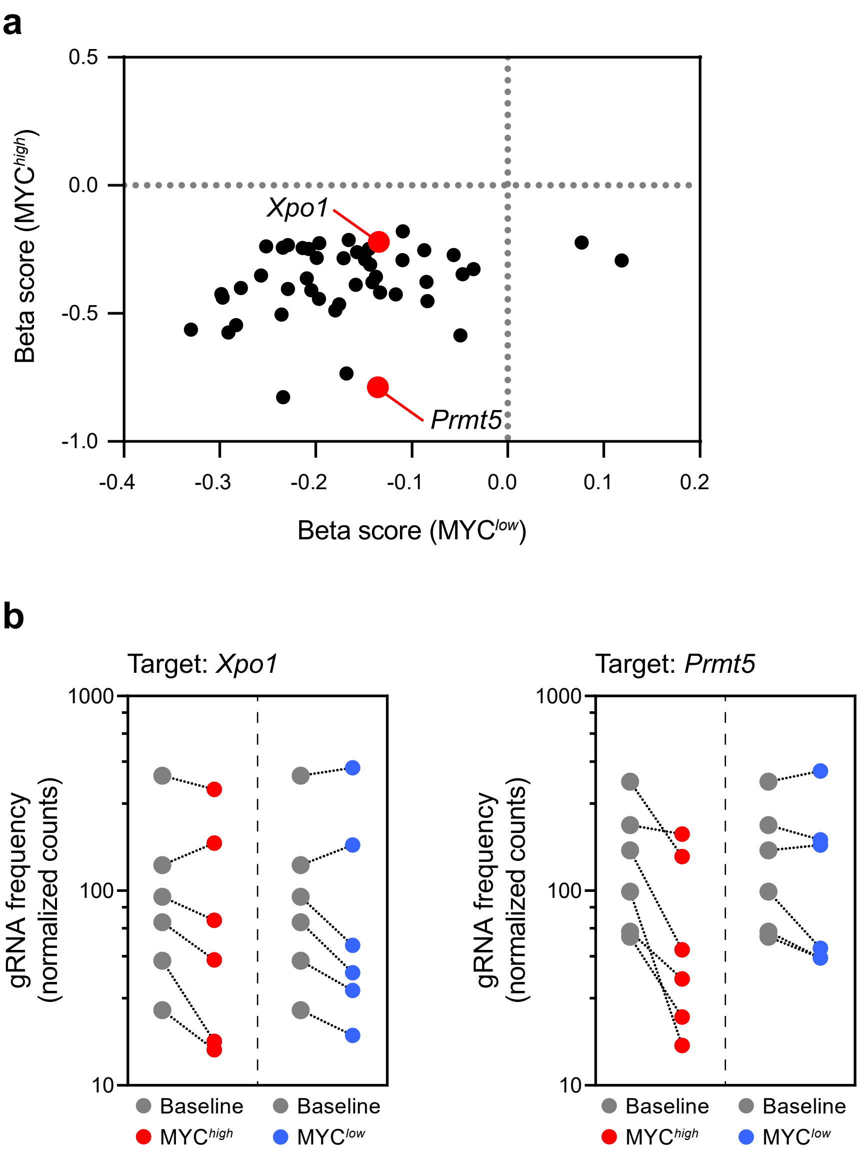

Supplementary Fig 6. Gene essentiality scores of MYC-SL RNA transport genes.
a, Scatter plot of Beta scores (gene essentiality scores) in the control (MYC^{low}; x-axis) versus MYC-SL (MYC^{high}; y-axis) condition. Shown are RNA transport genes that were MYC-SL and significantly upregulated by MYC. *Xpo1* and *Prmt5* are highlighted in red. **b,** Frequencies of gRNAs targeting *Xpo1* (left panel) and *Prmt5* (right panel). Normalized counts of six gRNAs per gene are shown at baseline, MYC^{high}, and MYC^{low} conditions. For this figure, source data are provided as a Source Data file.

Supplementary Figure 7

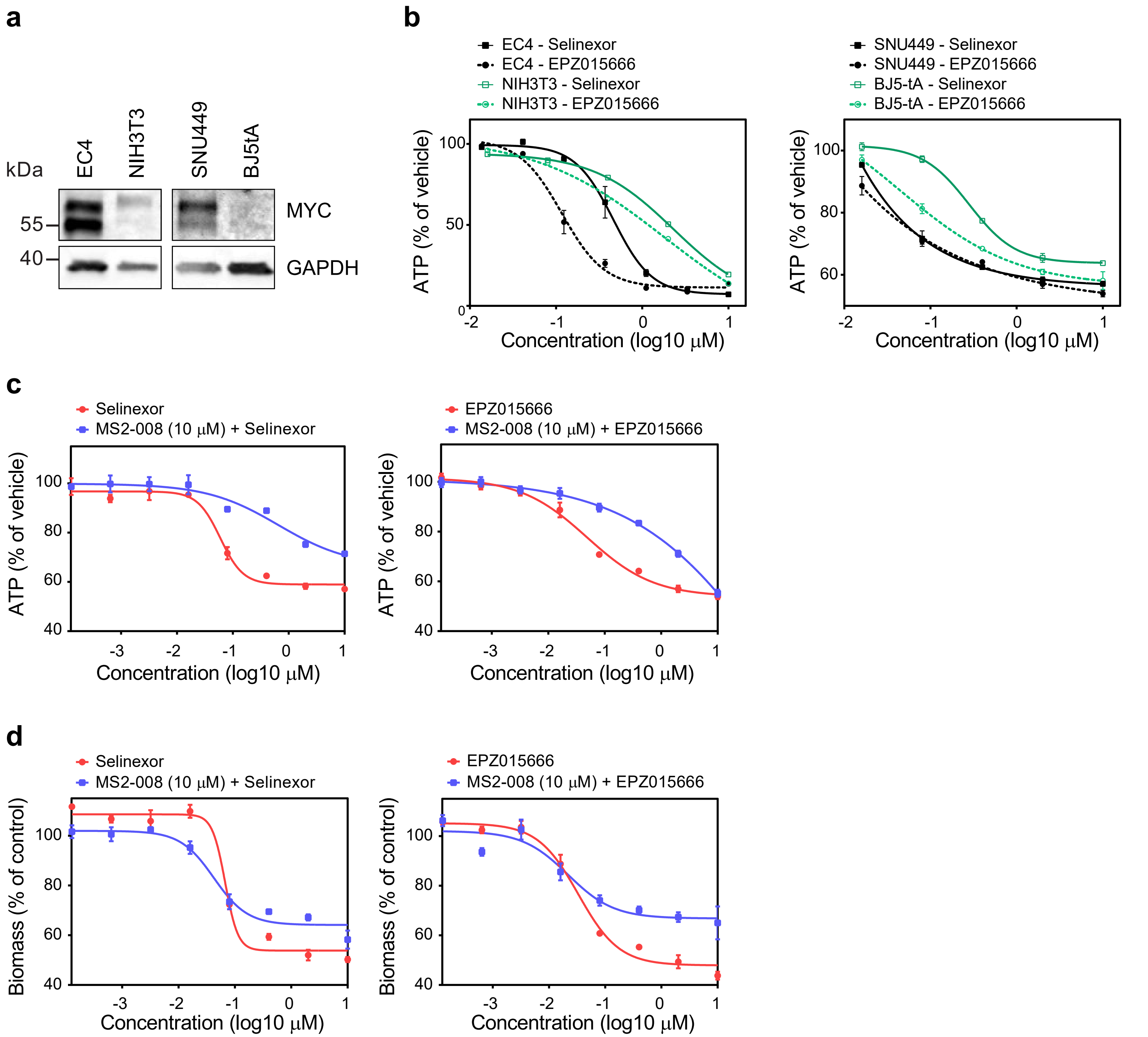

Supplementary Fig 7. *In vitro* efficacy studies of XPO1 and PRMT5 inhibitiors.
a, Western blot analysis of MYC expression in HCC (murine: EC4, human: SNU449) and fibroblast (murine: NIH3T3, human: BJ5tA) cell lines (n = 1). **b,** Effect of XPO1 inhibitor (Selinexor) and PRMT5 inhibitor (EPZ015666) on ATP levels in HCC cell lines (black symbols and curves) and fibroblasts (green symbols and curves). **c,d,** Impact of MYC activity on sensitivity to XPO1 or PRMT5 inhibition. SNU449 cells were treated with XPO1 inhibitor (left panel) or PRMT5 inhibitor (right panel) alone or in combination with the MYC activity inhibitor MS2-008. Efficacy of XPO1i and PRMT5i were assessed via measurement of ATP levels **(c)** and bio mass **(d)** using a Sulforhodamine B assay. Data points (**b-d**) are means of biological independent replicates/wells of one experiment, error bars are SEM. For this figure, source data are provided as a Source Data file.

Supplementary Figure 8

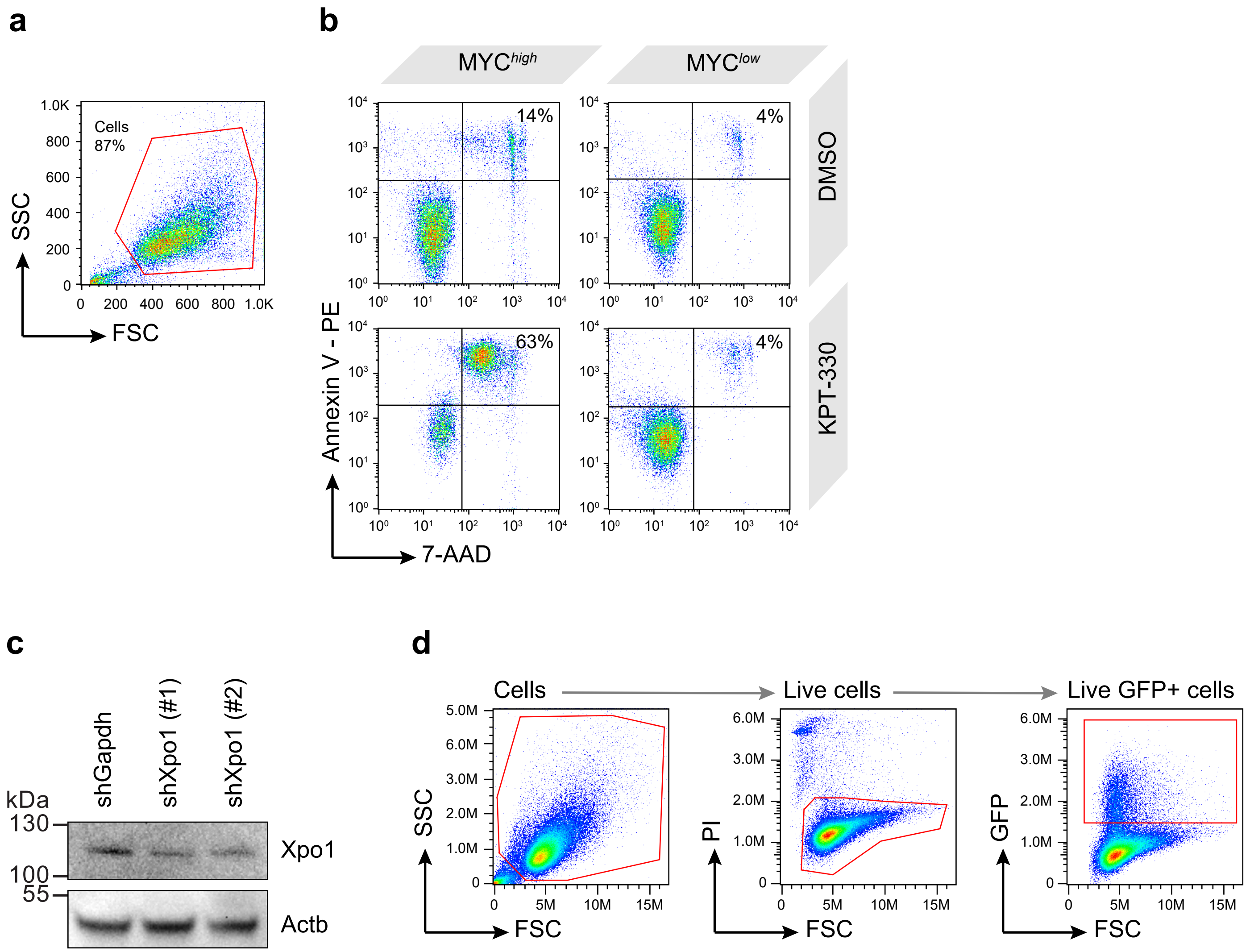

Supplementary Fig 8. Inhibition or downregulation of XPO1 in EC4 cells.
a-c, Flow cytometric analysis of EC4 cells expressing high or low MYC levels treated with XPO1 inhibitor. Cells were treated with KPT-330 (1 μM) or DMSO for 48 hours and collected for flow cytometric analysis after 7-AAD/Annexin V staining. For the MYC*low* condition, EC4 cells were treated with doxycycline (100 ng/ml). **a,** Cells were identified in forward scatter (FSC) versus sideward scatter (SSC). **b,** One respresentative plot of biologigal triplicates/wells is shown for each treatment condition. **c,** Western blot analysis of Xpo1 expression upon shRNA-mediated knockdown using two different Xpo1-targeting shRNAs. Gapdh-specific shRNA was used as negative control. Beta-Actin (Actb) served as loading control. Source data are provided as a Source Data file. **d,** Gating strategy for live GFP-positive cells for data presented in Figure 3f. PI: propidium iodide, GFP: green fluorescent protein.

Supplementary Figure 9

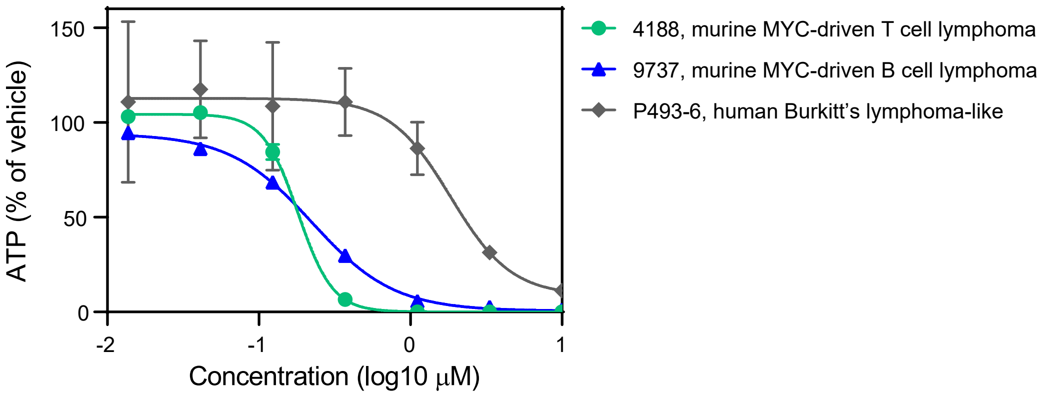

Supplementary Fig 9. Sensitivity of MYC-driven T cell and B cell lymphoma cells to XPO1 inhibition. 4188 cells (murine T cell lymphoma, green circles), 9737 cells (murine B cell lymphoma, blue triangles), and P493-6 cells (human Burkitt's lymphoma-like, grey diamonds) were treated for 48 hours with indicated concentrations of XPO1 inhibitor Selinexor. Cell survival was estimated by quantification of ATP levels by CellTiter-Glo Luminescent Cell Viability Assay. Data points are means of biological replicates (3 wells) of one experiment per indicated treatment, error bars are SEM. Source data are provided as a Source Data file.

Supplementary Figure 10

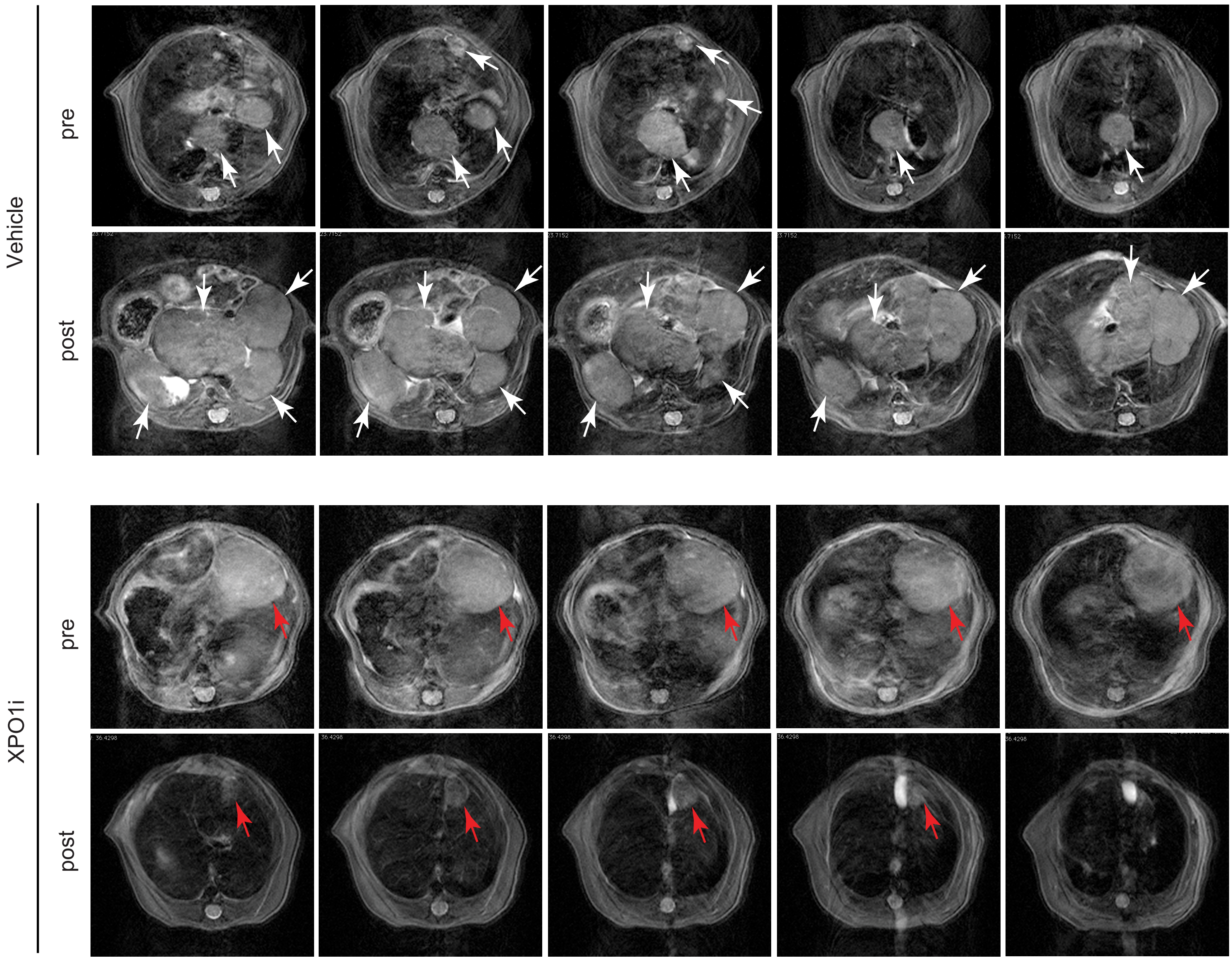

Supplementary Fig 10. Serial MRI images of primary hepatocellular carcinoma from *LAP-tTA/tet-O-MYC* mice.
Representative serial T2-weighted transverse images of tumor-bearing mice treated with vehicle control (upper panel) or with XPO1 inhibitor (XPO1i) Selinexor (lower panel). Both pre- and post-treatment (three doses) images are shown for a representative mouse from each treatement group. The tumor nodules are indicated by white (vehicle) or red (Selinexor) arrows. Part of the data is shown in Figure 4C.

Supplementary Figure 11

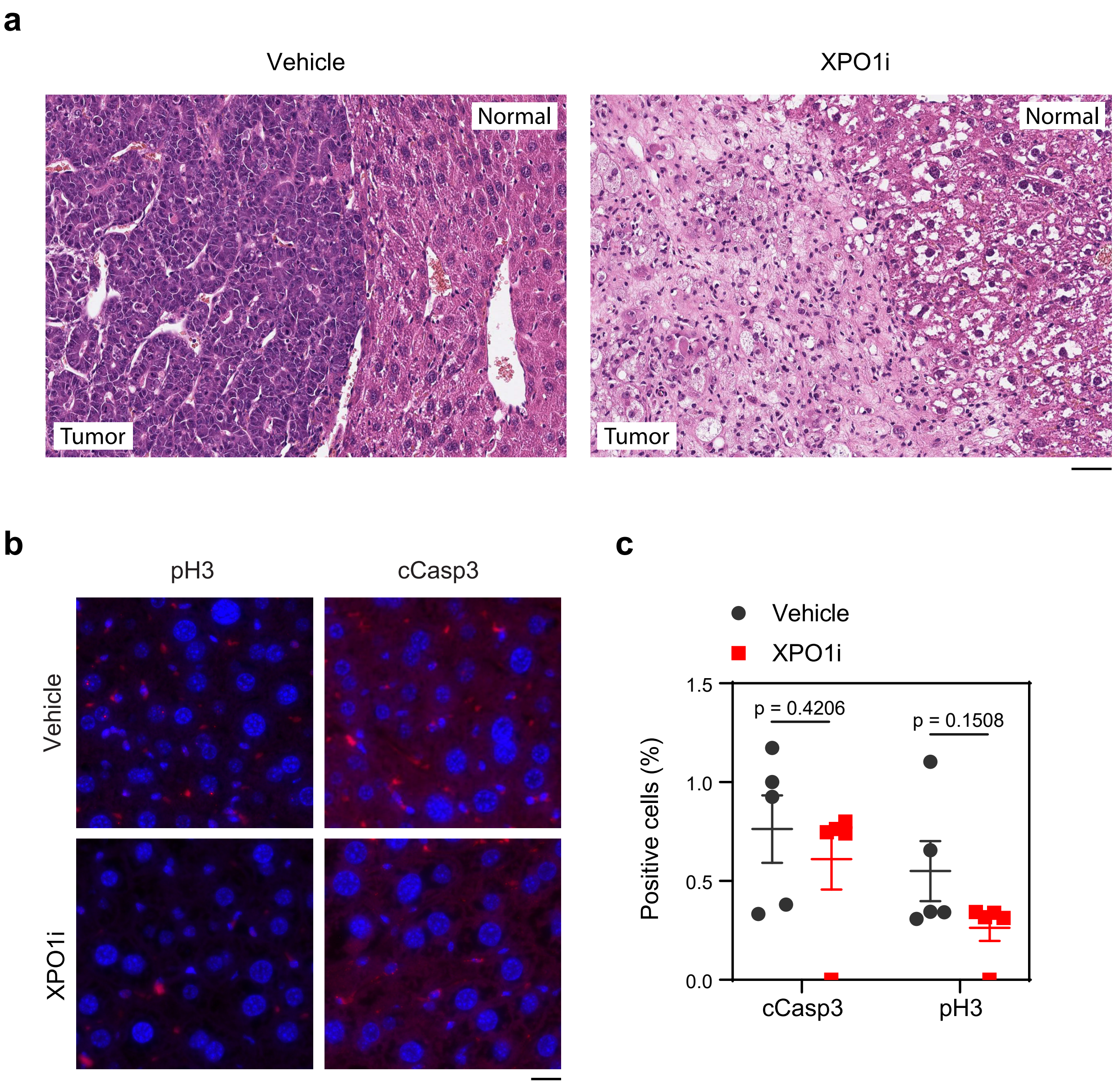

Supplementary Fig 11. Histological analysis of primary MYC-induced HCC treated with XPO1 inhibitor Selinexor.

a) Hematoxylin-eosin staining of primary hepatocellular carcinoma from LAP-tTA/tet-O-MYC mice treated with vehicle control (n = 3 mice) or Selinexor (n = 4 mice). Mice were treated with 3 doses per week and sacrificed on 7 days post treatment start. Both liver tumors (T) and the adjacent normal liver tissues (N) are shown for one representative mouse per condition. Scale bar = 50 μm. Images include regions shown in Figure 4d. **b)** Representative immunofluorescence images of tumor-adjacent normal liver tissue stained for phospho-histone H3 (pH3) and cleaved-caspase 3 (cCasp3) of mice described in a). Cell nuclei were counterstained with DAPI. Scale bar = 20 μm. **c)** Quantification of pH3- and cCasp3-positive cells in vehicle or XPO1 inhibitor (XPO1i)-treated tumor-adjacent normal liver tissue (n = 5 high-power fields (HPFs) of one representative mouse per treatment condition; lines show means, error bars are SEM; analyzed by two-tailed Mann-Whitney test). Source data are provided as a Source Data file.

Supplementary Figure 12

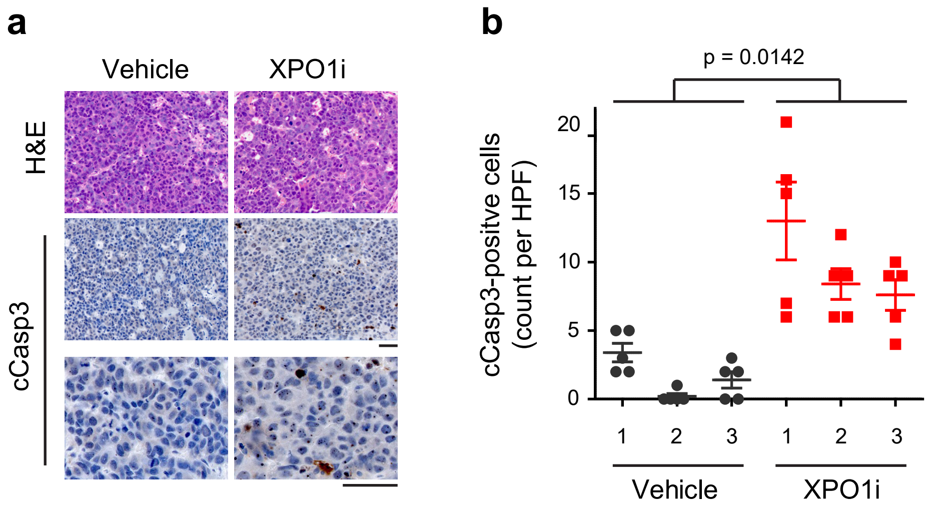

Supplementary Fig 12. Histological analysis of HCC PDX treated with XPO1 inhibitor Selinexor. **a,** Histology and immunohistochemistry (IHC) specific for cleaved caspase 3 (cCasp3) in PDX tumors treated with vehicle (n = 3 mice) or XPO1 inhibitor Selinexor (n = 3 mice). Representative hematoxylin-eosin (H&E) staining (top) and IHC staining (middle and bottom) of tumor tissue sections of one mouse per condition are shown. Scale bars = 50µm. **b,** Quantification of cCasp3-positive cells in tissue sections of PDX treated with vehicle (black circles) or XPO1 inhibitor Selinexor (red squares). cCasp3-positive cells were counted in five high power fields (HPFs) in tumor tissue sections from 3 mice per treatment. Data points show count per HPF, lines show mean counts for each mouse, error bars are SEM, p value from two-tailed Student's t-test. Source data are provided as a Source Data file.
