# Supplementary material for: Nuclear to cytoplasmic transport is a druggable dependency in MYC-driven hepatocellular carcinoma
Source: Nat Commun. 2024 Feb 1;15:963. doi: 10.1038/s41467-024-45128-y (PMC10834515; doi:10.1038/s41467-024-45128-y)
Supplement: Supplementary file 3 — Reporting Summary [file 41467_2024_45128_MOESM3_ESM.pdf]

nature portfolio

Corresponding author(s): Dean W. Felsher, MD PhD

Last updated by author(s): Oct 30, 2023

Reporting Summary

Nature Portfolio wishes to improve the reproducibility of the work that we publish. This form provides structure for consistency and transparency in reporting. For further information on Nature Portfolio policies, see our Editorial Policies and the Editorial Policy Checklist.

Statistics

For all statistical analyses, confirm that the following items are present in the figure legend, table legend, main text, or Methods section.

n/a	Confirmed	
☐	☒	The exact sample size (*n*) for each experimental group/condition, given as a discrete number and unit of measurement
☐	☒	A statement on whether measurements were taken from distinct samples or whether the same sample was measured repeatedly
☐	☒	The statistical test(s) used AND whether they are one- or two-sided<br>*Only common tests should be described solely by name; describe more complex techniques in the Methods section.*
☒	☐	A description of all covariates tested
☐	☒	A description of any assumptions or corrections, such as tests of normality and adjustment for multiple comparisons
☐	☒	A full description of the statistical parameters including central tendency (e.g. means) or other basic estimates (e.g. regression coefficient) AND variation (e.g. standard deviation) or associated estimates of uncertainty (e.g. confidence intervals)
☐	☒	For null hypothesis testing, the test statistic (e.g. *F*, *t*, *r*) with confidence intervals, effect sizes, degrees of freedom and *P* value noted<br>*Give P values as exact values whenever suitable.*
☒	☐	For Bayesian analysis, information on the choice of priors and Markov chain Monte Carlo settings
☒	☐	For hierarchical and complex designs, identification of the appropriate level for tests and full reporting of outcomes
☐	☒	Estimates of effect sizes (e.g. Cohen's *d*, Pearson's *r*), indicating how they were calculated

Our web collection on statistics for biologists contains articles on many of the points above.

Software and code

Policy information about availability of computer code

Data collection	Instrument specific acquisition software is described in Methods.
Data analysis	MAGeCK (v0.5.9.2), DESeq2 (v1.22.2), R (v3.6.1), singscore (v1.14.0), RankProd (v3.14.0), STAR (v2.5.4b), GSEA (v4.1.0), ImageJ (v1.47k), OsiriX Lite (v12.5.0), FlowJo (v10.8.1), MetaMorph (v7.8), GraphPad Prism (v9.3.1). All custom code used in this study is deposited in https://github.com/Yenaled/felsher.

For manuscripts utilizing custom algorithms or software that are central to the research but not yet described in published literature, software must be made available to editors and reviewers. We strongly encourage code deposition in a community repository (e.g. GitHub). See the Nature Portfolio guidelines for submitting code & software for further information.

Data

Policy information about availability of data

All manuscripts must include a data availability statement. This statement should provide the following information, where applicable:

- Accession codes, unique identifiers, or web links for publicly available datasets
- A description of any restrictions on data availability
- For clinical datasets or third party data, please ensure that the statement adheres to our policy

All raw sequencing data generated in this study have been deposited in Gene Expression Omnibus and are available in Series GSE205132. Sub-series GSE205130

contains RNA sequencing data, sub-series GSE205131 contains genome-wide CRISPR/Cas9 screening data. Third party datadsets used in this study are publically available and sources have been cited.

Human research participants

Policy information about studies involving human research participants and Sex and Gender in Research.

Reporting on sex and gender	Sex and gender have not been considered in this study. Sex- or gender-based analyses were not performed due to limited sample size. Two patient samples were used to generate patient-derived xenografts.
Population characteristics	NA
Recruitment	NA
Ethics oversight	Tissue sample collection following informed consent was approved by the Stanford University Institutional Review Board.

Note that full information on the approval of the study protocol must also be provided in the manuscript.

Field-specific reporting

Please select the one below that is the best fit for your research. If you are not sure, read the appropriate sections before making your selection.

☒ Life sciences ☐ Behavioural & social sciences ☐ Ecological, evolutionary & environmental sciences

For a reference copy of the document with all sections, see nature.com/documents/nr-reporting-summary-flat.pdf

Life sciences study design

All studies must disclose on these points even when the disclosure is negative.

Sample size	Sample sizes were determined empirically based on pilot experiments or similar studies. No statistical method was used to determine sample size. For CRISPR/Cas9 screen synthetic lethal screen, one sample was used for each condition (baseline, MYC high, MYC low). For RNA-seq experiments using EC4 cells, three replicates were used per sample; for RNA-seq experiments using murine tissue samples, two samples were used for normal, three samples for MYC low, and 6 samples for MYC high. Cell viability and fitness experiments were performed in triplicates. In vivo experiments were performed with at least four mice per group. Sample sizes are indicated for each experiment. We determined these sample sizes to be sufficient owing to variability in sample groups.
Data exclusions	No data were excluded.
Replication	The genome-wide CRISPR screen was performed once. RNA-seq experiments contain independent biological replicates. Sequencing was performed once. Analysis of sequencing data was performed by two investigators independently and analysis results were successfully reproduced. Cell-based in vitro assays contain technical triplicates and were repeated at least twice with comparable results confirming reproducibility of results. Immunofluorescence and immunohistochemistry stainings were performed once on independent biological replicates (number of biological replicates is indicated in figure legends). Western blots were performed once. In vivo experiments were performed once with indicated number of animals.
Randomization	Mice bearing MYC-driven HCC as confirmed by magnetic resonance imaging were randomized into treatment groups (vehicle, inhibitor). Mice bearing subcutaneous PDX tumors were randomized into treatment groups (vehicle, inhibitor). For all in vitro experiments, cells were randomly allocated into treatment groups.
Blinding	Determination of HCC tumor volumes based on MRI images and PDX tumor size measurements by caliper were performed by blinded investigators. For vitro experiments, the investigators were either blinded during treatment group allocation or during data acquisition/ analysis.

Reporting for specific materials, systems and methods

We require information from authors about some types of materials, experimental systems and methods used in many studies. Here, indicate whether each material, system or method listed is relevant to your study. If you are not sure if a list item applies to your research, read the appropriate section before selecting a response.

Materials & experimental systems

n/a	Involved in the study
	☒ Antibodies
	☒ Eukaryotic cell lines
☒	☐ Palaeontology and archaeology
	☒ Animals and other organisms
☒	☐ Clinical data
☒	☐ Dual use research of concern

Methods

n/a	Involved in the study
☒	☐ ChIP-seq
	☒ Flow cytometry
☒	☐ MRI-based neuroimaging

Antibodies

Antibodies used

Anti-MYC clone 9E10 (1:5000, Millipore-Sigma, M4439), Anti-MYC clone Y69 (1:1000, Abcam, Ab32072), Anti-Exportin-1 clone D6V7N (1:1000, Cell Signaling Technology, 46249), Anti-GAPDH clone 14C10 (1:5000, Cell Signaling Technology, 2118), Anti-alpha Tubulin clone DM1A (1:2000, Millipore-Sigma, T9026), Anti-beta Actin (1:1000, Cell Signaling Technology, 4967), Anti-rabbit IgG IRDye800CW (1:10000, Licor, 926-32211), Anti-mouse IgG IRDye680RD (1:10000, Licor, 926-68070), Anti-rabbit IgG-AP (1:5000, Invitrogen, G-21079), Anti-mouse IgG/IgM-AP (1:5000, Invitrogen, 31330), Anti-MYC clone EP121 (1:150, Millipore-Sigma, 395R), Anti-cleaved Caspase-3 (Asp175) (1:100, Cell Signaling Technology, 9661), Biotinylated anti-rabbit IgG (1:500, Vector Laboratories, BA-1000-1.5), Anti-phospho Histone-3 (Ser10) (1:200, Cell Signaling Technology, 9701), Anti-rabbit IgG AlexaFluor568 (1:400, Invitrogen, A-11011).

Validation

https://www.sigmaaldrich.com/US/en/product/sigma/m4439
https://www.abcam.com/products/primary-antibodies/c-myc-antibody-y69-chip-grade-ab32072.html
https://www.sigmaaldrich.com/US/en/product/sigma/395r1
https://www.cellsignal.com/products/primary-antibodies/exportin-1-crm1-d6v7n-rabbit-mab/46249
https://www.cellsignal.com/products/primary-antibodies/phospho-histone-h3-ser10-antibody/9701
https://www.cellsignal.com/products/primary-antibodies/cleaved-caspase-3-asp175-antibody/9661
https://www.cellsignal.com/products/primary-antibodies/gapdh-14c10-rabbit-mab/2118
https://www.sigmaaldrich.com/US/en/product/sigma/T9026
https://www.thermofisher.com/antibody/product/Goat-anti-Rabbit-IgG-H-L-Cross-Adsorbed-Secondary-Antibody-Polyclonal/A-11011
https://vectorlabs.com/products/biotinylated-goat-anti-rabbit-igg/
https://www.licor.com/bio/reagents/irdye-800cw-goat-anti-rabbit-igg-secondary-antibody
https://www.licor.com/bio/reagents/irdye-680rd-goat-anti-mouse-igg-secondary-antibody
https://www.thermofisher.com/antibody/product/Goat-anti-Rabbit-IgG-H-L-Cross-Adsorbed-Secondary-Antibody-Polyclonal/G-21079
https://www.thermofisher.com/antibody/product/Goat-anti-Mouse-IgG-IgM-H-L-Cross-Adsorbed-Secondary-Antibody-Polyclonal/31330

Eukaryotic cell lines

Policy information about cell lines and Sex and Gender in Research

Cell line source(s)

Human cell lines SNU-449, BJ5ta, and murine cell line NIH3T3 were obtained from ATCC, 293FT cells were obtained from Thermo Fisher Scientific. Murine cell lines EC4, 4188, and 9737 were previously generated in the Felsher laboratory from MYC-driven tumors. P493-6 cells were kindly provided by Prof. Chi V. Dang (Ludwig Institute for Cancer Research, Johns Hopkins University School of Medicine, Baltimore, USA).

Authentication

Cell lines were not authenticated.

Mycoplasma contamination

All cell lines used in this study tested negative for Mycoplasma.

Commonly misidentified lines (See ICLAC register)

No commonly misidentified cell lines were used in this study.

Animals and other research organisms

Policy information about studies involving animals; ARRIVE guidelines recommended for reporting animal research, and Sex and Gender in Research

Laboratory animals

LAP-tTA/tet-O-MYC/FVB/N and NSG mice were used for efficacy studies.

Wild animals

The study did not involve wild animals.

Reporting on sex

Findings apply only to males. In the LAP-tTA/tet-O-MYC/FVB/N mouse model of HCC, only male mice develop liver tumors.

Field-collected samples

The study did not involve samples collected from the field.

Ethics oversight

Animal experiments described in this study have been approved by Administrative Panel on Laboratory Animal Care at Stanford

Ethics oversight | University and comply with all federal and state regulations governing the humane care and use of laboratory animals, including the USDA Animal Welfare Act, and Stanford's Assurance of Compliance with PHS Policy on Humane Care and Use of Laboratory Animals.

Note that full information on the approval of the study protocol must also be provided in the manuscript.

Flow Cytometry

Plots

Confirm that:

☒ The axis labels state the marker and fluorochrome used (e.g. CD4-FITC).

☒ The axis scales are clearly visible. Include numbers along axes only for bottom left plot of group (a 'group' is an analysis of identical markers).

☒ All plots are contour plots with outliers or pseudocolor plots.

☒ A numerical value for number of cells or percentage (with statistics) is provided.

Methodology

Sample preparation	Murine MYC-driven HCC cells were treated as described in vitro. Cells in supernatant and adherent cells were collected for flow cytometric analysis.
Instrument	Samples were anlyzed using a BD FACScan or Accuri C6 Plus system.
Software	BD Cell Quest or BD Accuri C6 Plus software was used for data collection. FlowJo software was used for data analysis.
Cell population abundance	No cell sorting was performed in this study.
Gating strategy	Cells were identified in a FSC/SSC gate. Single cells were gated on using a FSC-A/FSC-H gate. Live (Propidium iodide negative) cells were identified in a FL-3 (PI)/FSC-H gate. GFP-negative cells were used to determine gate position to identify GFP-positive cells.

☒ Tick this box to confirm that a figure exemplifying the gating strategy is provided in the Supplementary Information.
