# Supplementary material for: Nuclear to cytoplasmic transport is a druggable dependency in MYC-driven hepatocellular carcinoma
Source: Nat Commun. 2024 Feb 1;15:963. doi: 10.1038/s41467-024-45128-y (PMC10834515; doi:10.1038/s41467-024-45128-y)
Supplement: Supplementary file 4 — Description of Additional Supplementary Files [file 41467_2024_45128_MOESM4_ESM.pdf]

Description of Additional Supplementary Files

Supplementary Data 1: Differential gene expression analysis (normalized counts). MYC-driven murine HCC cell line (EC4) pre (MYC^{high}) and seven days post (MYC^{low}) inactivation of MYC transgene expression (see Supplementary Figure 3b-e).

Supplementary Data 2: CRISPR-Cas9 screen essentiality scores.

Supplementary Data 3: Functional gene enrichment analysis of 1808 MYC synthetic-lethal genes (see Figure 1d).

Supplementary Data 4: Rank product analysis of gene essentiality scores in human HCC cell lines (CCLE).

Supplementary Data 5: Functional gene enrichment analysis of 342 genes in human HCC cell lines and murine HCC with high MYC expression (see Figure 1e).

Supplementary Data 6: Differential gene expression analysis (log2FoldChange). Primary murine HCC tumors pre (MYC^{high}) and post (MYC^{low}) inactivation of MYC transgene expression (see Figure 3a-c).

Supplementary Data 7: Differential gene expression analysis (log2FoldChange). Primary murine HCC tumors (MYC^{high}) and normal liver (see Figure 3c).

Supplementary Data 8: Functional gene enrichment analysis of 516 MYC synthetic-lethal that are upregulated in primary murine MYC^{high} tumors (see Figure 3b).
